# Longitudinal associations between prenatal internalizing symptoms and mindfulness traits with postnatal bonding difficulties

**DOI:** 10.1007/s00737-024-01518-1

**Published:** 2024-09-24

**Authors:** Julia Garon-Bissonnette, Christian A. L. Bean, Emilia F. Cárdenas, Maya Jackson, George Abitante, Kathryn L. Humphreys, Autumn Kujawa

**Affiliations:** 1 Department of Psychology and Human Development, Vanderbilt University, Nashville, TN USA; 2 Department of Special Education, Vanderbilt University, 230 Appleton Place, Nashville, TN USA

**Keywords:** Bonding, Postpartum, Multilevel modelling, Mindfulness, Internalizing

## Abstract

**Purpose:**

Mothers’ reported connection, or bond, with their infants develops across the early postnatal period and is relevant to mother and offspring functioning. Little is known, however, about early predictors of bonding difficulties over time. The present study examined prenatal anxiety, depressive symptoms, and trait mindfulness and variation in bonding difficulties in mothers across the first two months postnatal.

**Methods:**

Participants were 120 pregnant women (*M*_*age*_=31.09 years, *SD* = 4.81; 80% White). Measures of anxiety, depression, and five facets of mindfulness were administered mid-pregnancy (approximately 20 weeks gestation) and bonding difficulties were assessed every two weeks from approximately 1 to 7 weeks postnatal.

**Results:**

Using multilevel modeling to account for within-person repeated assessments, we found an inverted U-shaped pattern across time such that bonding difficulties initially worsened before improving around five weeks postnatal. Prenatal anxiety and depressive symptoms were longitudinally associated with greater bonding difficulties overall and were unrelated to the trajectory of change. The mindfulness facets of acting with awareness and being nonjudging of one’s own experience were longitudinally associated with less bonding difficulties overall, weaker initial increases in bonding difficulties, and earlier improvements.

**Conclusions:**

Prenatal anxiety and depression may be risk factors for bonding difficulties that are persistent across the early postnatal period. In contrast, mindfulness tendencies before childbirth, specifically acting with awareness and being nonjudging towards oneself, may support early feelings of bonding over time.

**Supplementary Information:**

The online version contains supplementary material available at 10.1007/s00737-024-01518-1.

## Article highlights

Feelings of bonding are stable yet dynamic during early postnatal, with increased difficulties between three and five weeks postnatal.

After initially increasing following birth, maternal-reported bonding difficulties declined from five to seven weeks postnatal.

Higher prenatal internalizing symptoms link to increased bonding difficulties across time.

Some facets of the mindfulness stance during pregnancy link to lower bonding difficulties over time and earlier improvements in bonding.

**Supplementary Information:**

The online version contains supplementary material available at 10.1007/s00737-024-01518-1.

Most people directly or indirectly experience pregnancy at some point in their life, including being pregnant or expecting a child themselves, and eventually become parents (e.g., 85% of women in the US are estimated to give birth by age 50; U.S. Census Bureau 2020). Early during pregnancy, pregnant individuals begin to develop a bond with their future child (Benoit et al. 1997; Cranley 1993), and the strength of this bond grows over time (Roth et al. 2021), notably after meeting their newborn (de Cock et al. 2016). In the scientific literature, this feeling of connection or “bond” to the (unborn) child is often referred to as bonding (Bicking Kinsey and Hupcey 2013; Cranley 1993; Klaus and Kennell 1976). Bonding is typically measured using self-reported caregiver *feelings* of connection toward their infants and should not be conflated with caregiver–infant attachment (Bicking Kinsey and Hupcey 2013).

Caregivers’ feelings of connecting with their infants are posited to develop over time, although there are individual differences in the shape of the trajectories of bonding. Studies have shown moderate stability in reported bonding from pregnancy to 6 and 24 months (de Cock et al. 2016) as well as from 8 to 14 weeks postnatal (Döblin et al. 2023) in both mothers and fathers (although we acknowledge that pregnant and birthing individuals have different gender identities, we use women and mothers—or men and fathers—for consistency with past literature). Although relatively stable, maternal reported bonding increases from 1 to 6 months (Roth et al. 2021). One study evaluating late pregnancy to 24 months documented most change between late pregnancy and six months postnatal (de Cock et al. 2016), and another focusing on six weeks to six months postnatal documented most change between six weeks and four months postnatal (Muzik et al. 2013). Given early maternal-reported bonding holds predictive value over later functioning (Faisal-Cury et al. 2021), these results highlight the importance of a close examination of bonding across the early postnatal period. However, the lack of repeated assessments in most studies limits our understanding of how feelings of connection evolve during the very early postnatal weeks.

Given that maternal-reported bonding is associated with maternal adaptation (Figueiredo and Costa 2009), observed caregiving behaviors (Muzik et al. 2013), and child development (Rusanen et al. 2024), it is crucial to identify predictors of bonding difficulties. Most research explored risk factors such as pre- and postnatal internalizing symptoms (i.e., depression and anxiety), personality style, and health risks (Dubber et al. 2015; Farré-Sender et al. 2018; Tichelman et al. 2019). Regarding internalizing symptoms, some studies documented that prenatal depression, but not anxiety, explained variance in maternal-reported bonding (Dubber et al. 2015), whereas others have found the opposite and highlighted the role of anxiety over depression (Farré-Sender et al. 2018). In addition to these discrepant findings, most studies have focused on a fixed moment in time and used cross-sectional designs, limiting our ability to understand the temporal relations between perinatal internalizing symptoms and bonding difficulties.

Studies evaluating factors that promote bonding and hold the potential for informing preventive interventions are rare. One promising factor is mindfulness, a multifaceted construct including tendencies to act with awareness, focus on the present moment, and accept one’s own experiences in a nonjudgmental and nonreactive manner (Kabat-Zinn 2015). A recent study found that prenatal tendencies to act with awareness, but not other facets of mindfulness, were associated with postnatal feelings of investment towards babies over other risk factors for caregiving (i.e., depression, anxiety, and self-criticism; Brassel et al. 2020). However, this evidence is limited by a small sample (*n* = 32) and a single assessment of maternal feelings of investment, a proxy of bonding, rather late in the development of the caregiver–child relationship (at child age 18 months).

The present study aimed to examine trajectories of change in maternal bonding difficulties across four time points in the first two months postnatal and evaluate early predictors (anxiety, depression, and mindfulness traits) of bonding difficulties. We tested for linear and quadratic time trends and did not formulate a priori hypotheses regarding how bonding difficulties would develop across the first two months postnatal given the exploratory nature of this aim. Overall, we expected that higher prenatal anxiety and depression would be associated with increased bonding difficulties and that higher trait mindfulness would promote feelings of bond. We also hypothesized that increased prenatal depression and anxiety would be associated with a stronger linear effect of time (i.e., bonding difficulties over time). In contrast, we hypothesized that greater prenatal mindfulness would predict lower bonding difficulties. We did not formulate a priori hypotheses about which specific facets of mindfulness would be associated with bonding.

## Materials and methods

### Participants and procedure

Pregnant individuals were recruited through prenatal care clinics, advertisements distributed throughout an academic medical center and the broader community, and social media between September 2020 and April 2022 to participate in a longitudinal study on peripartum depression. Inclusion criteria were being currently pregnant, eighteen years or older, and proficient in English. Exclusion criteria were being older than forty years old, having diagnoses of bipolar disorder, psychosis, or borderline personality disorder, and carrying multiples or a fetus with a known congenital disorder. The study received ethical approval from the affiliated university’s Institutional Review Board.

One hundred and twenty pregnant individuals participated at the initial assessment. The power calculations for parent study aims determined > 0.80 power with a sample size of 100, and we over-enrolled to account for attrition and missingness. Participants all identified as women and were primarily White (80%), non-Hispanic/Latinx (91%), and married or in domestic partnerships (85%; Table 1). We oversampled participants at heightened risk for peripartum depression. Participants completed prenatal assessments around 20 weeks’ gestation, four equally spaced surveys from 1 to 7 weeks postnatal (wk1-wk7) and postnatal assessments around 9 weeks postnatal (see Supplementary Methods for detailed information on depression risk assessment and study timepoints and procedure).

**Table 1:** Demographics characteristics of the sample^a^

Characteristic	*M*	*SD*
Maternal age (years)	30.75	4.99
Gestational weeks (prenatal)	21.80	3.16
Postnatal weeks (postnatal)	9.23	2.53
	***n***	**%**
Parity		
Primiparous	72	61.6
Multiparous	42	35.9
Missing	3	2.6
Ethnicity		
Hispanic or Latina/e/o/x	11	9.4
Not Hispanic or Latina/e/o/x	106	90.6
Race		
White	93	79.5
Black/African American	12	10.3
Asian	3	2.6
Multiracial	3	2.6
Other	6	5.1
Educational attainment		
Some college or less	20	17.1
Associate or bachelor’s degree or higher	97	82.9
Relationship status		
Married or domestic partnership	99	84.6
Single and never married or divorced	18	15.4
Annual household income		
$30,000 or less	9	7.7
$30,001-$60,000	20	17.2
$60,001-$90,000	26	22.4
$90,001-$150,000	35	30.2
Greater than $150,000	26	22.4
Missing	1	0.8

*Note* Demographics were collected at the prenatal assessment

^a^ Demographics were available for 117 participants

### Measures

*Pre-and postnatal anxiety* was measured using the Generalized Anxiety Disorder Assessment (GAD-7; Spitzer et al. 2006), a self-reported screening tool. The GAD-7 has 7 items evaluated on a 4-point Likert scale from 0 (*Not at all*) to 3 (*Nearly every day*). Higher scores indicate higher severity of generalized anxiety symptoms (range = 0–21). Internal consistency was excellent (prenatal α = 0.90 and postnatal α = 0.89).

*Pre-and postnatal depressive symptoms* were measured using the General Depression subscale of the Inventory of Depression and Anxiety Symptoms (IDAS; Watson et al. 2007), a self-report of past two weeks depressive symptoms. The scale has 20 items evaluated on a 5-point Likert scale from 1 (*Not at all*) to 5 (*Extremely*), with higher scores indicating more symptoms (range = 20–100). Internal consistency was good to excellent (prenatal α = 0.92 and postnatal α = 0.87).

*Mindfulness disposition* during pregnancy was measured using the Five Facets Mindfulness Questionnaire (FFMQ; Baer et al. 2006). The FFMQ has 39 items evaluated on a 5-point Likert scale from 1 (*Never or very rarely true*) to 5 (*Very often or always true*) and measures five distinct facets of mindfulness: (1) observing (attending to sensations, perceptions, and feelings), (2) describing (labeling experiences), (3) acting with awareness (being non-distracted; avoiding automatic pilot), (4) nonjudging (accepting thoughts and feelings), and (5) nonreacting (refraining from impulsive reactions). Internal consistency was acceptable to excellent (observing α = 0.80, describing α = 0.93, acting with awareness α = 0.91, nonjudging α = 0.93, and nonreacting α = 0.74).

*Maternal bonding difficulties* from 1 to 7 weeks postnatal were assessed using the Postpartum Bonding Questionnaire (PBQ; Brockington et al. 2001), a self-reported measure assessing difficulties in caregivers’ perception of their bond with their infant. The PBQ has 25 items rated on a 6-point Likert scale from 0 (*Always*) to 5 (*Never*). We used the general factor of difficulties in bonding (12 items), with higher scores indicating increased bonding difficulties (range = 0–35). Internal consistency was acceptable to good across the four surveys (α_s_ between 0.74 and 0.83).

### Data analytic plan

We estimated growth curves using a multilevel modeling approach to test for linear and quadratic trends in bonding difficulties over time. Analyses were conducted in Stata version 17 (StataCorp 2021) and data visualization was conducted in R version 4.1.3 (R Core Team 2022). We first examined growth curves of bonding difficulties in unconditional models, without any predictors except time, using maximum likelihood approximation. Three potential models were considered: intercept-only, intercept and linear trend of time, and intercept and both linear and quadratic trends of time. Model fit was assessed via a comparison of nested-2 log-likelihood functions (Curran et al. 2010). All models featured a random slope effect of time and an unstructured random effects covariance matrix. The linear-trend only model did not statistically significantly improve model fit over the intercept-only model (Δχ^2^(3) = 6.91, *p =* .075). However, the model including both linear and quadratic trends improved model fit over the linear-trend (Δχ^2^(1) = 9.67, *p =* .002) and the intercept-only (Δχ^2^(4) = 16.58, *p =* .002) models and was thus chosen as the preferred model.

**Fig. 1:**
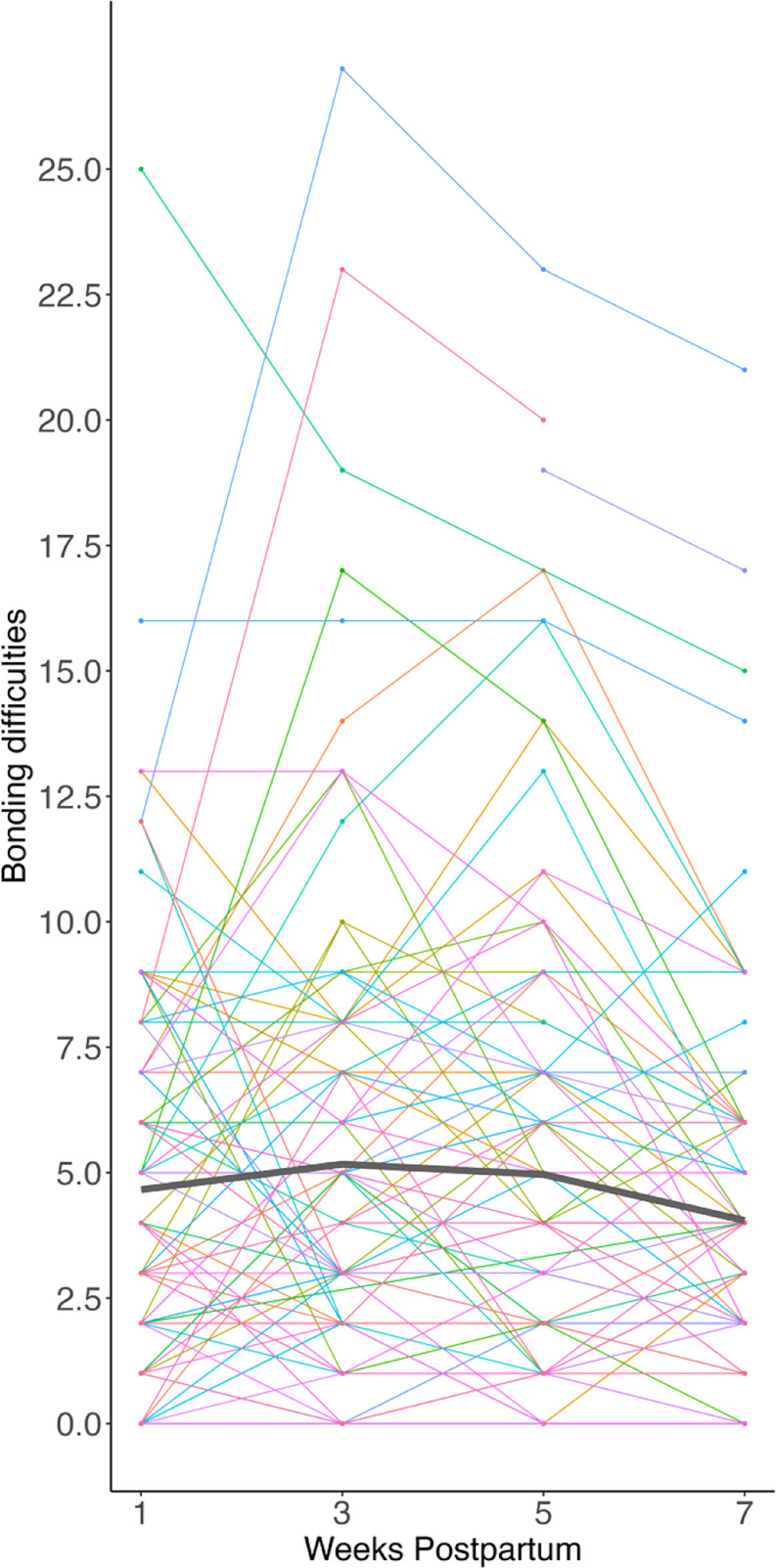
Curvilinear growth in maternal bonding difficulties across the first seven weeks postnatal. *Note* Bonding difficulties were present initially (i.e., intercept different than zero) and worsened before then improving around 5 weeks. Colored lines represent individual trajectories of feelings of bonding across four timepoints from 1 to 7 weeks postnatal. The thick gray line indicates the average curvilinear growth across all participants

In the unconditional model, the intercept was greater than zero (π_oj_ = 4.66, *z* = 10.23, *p* < .001, 95% CI [3.77, 5.55]). The linear trend of time was positive and statistically significant (π_1j_ = 0.86, *z* = 2.40, *p* = .016, 95% CI [0.16, 1.57]) and the quadratic effect of time was also statistically significant but negative (π_2j_= − 0.36, *z*= − 3.14, *p* = .002, 95% CI [− 0.58, − 0.13]). Thus, overall, bonding difficulties showed a quadratic trajectory of change across the postnatal period (Fig. 1).

We next examined prenatal depression, anxiety, and mindfulness. Each predictor was added as a time-invariant covariate to estimate its effect on the intercept (i.e., mean bonding difficulties). Potential interactions between each predictor and the linear and quadratic effects of time were examined in a second step. For instance, the following equation includes prenatal depression as a predictor of the intercept (β_10_) and moderator of the linear (β_11_) and quadratic (β_21_) trends:

$$\begin{aligned}\text{Level}\,{1:}\quad{Bonding}_{ij}&={\pi}_{0j}+{\pi}_{1j}\left({Time}_{ij}\right)\\ &\quad+{\pi}_{2j}\left({Time}_{ij}*{Time}_{ij}\right)+{e}_{ij}\end{aligned}$$

$$\text{Level}\,{2:}\quad{\pi}_{0j}={\beta}_{00}+{\beta}_{01}\left({Depression}_{j}\right)+{r}_{0j}$$

$$\:{\pi}_{1j}={\beta}_{10}+{\beta}_{11}\left({Depression}_{j}\right)+{r}_{1j}$$

$$\:{\pi}_{2j}={\beta}_{20}+{\beta}_{21}\left({Depression}_{j}\right)$$

A separate conditional growth model was estimated for each predictor: one with depression, one with anxiety, and one with each mindfulness facet. All were estimated using restricted maximum likelihood approximation. Time was centered at time 1 (1 week postnatal) and predictor variables were grand-mean centered. Sensitivity analyses were conducted including internalizing symptoms at 8 weeks postnatal as covariates in the full models with prenatal symptoms as predictors.

## Results

### Missing data analyses

One hundred and two participants (87.2%) provided bonding scores at 1 week postnatal, 99 (84.6%) at 3 weeks, 97 (82.9%) at 5 weeks, and 99 (84.6%) at 7 weeks postnatal. Participants completed 3.82 biweekly postnatal surveys on average. Participants with complete and partial data did not differ in ethnicity (χ^2^(1) = 0.85, *p* = .357), race (χ^2^(3) = 3.79, *p* = .285), education (χ^2^(5) = 3.56, *p* = .615), employment (χ^2^(5) = 1.28, *p* = .937), relationship status (χ^2^(1) = 0.36, *p* = .549), annual income (χ^2^(6) = 1.74, *p* = .942), mode of delivery (χ^2^(1) = 0.09, *p* = .765), fetal distress during delivery (χ^2^(1) = 0.19, *p* = .665), or infant sex (χ^2^(1) = 0.42, *p* = .517). Table 2 shows descriptive statistics and associations between variables.

**Table 2:** Means, standard deviations, and pairwise, bivariate correlations among study variables and demographics

	*M* (*SD*)	1	2	3	4	5	6	7	8	9	10	11	12	13
1. Prenatal IDAS	36.47 (11.04)	–												
2. Postnatal IDAS	36.59 (8.64)	0.57**	–											
3. Prenatal GAD-7	4.18 (4.11)	0.85**	0.46**	–										
4. Postnatal GAD-7	3.68 (3.97)	0.51**	0.76**	0.50**	–									
5. FFMQ Observe	26.55 (5.32)	0.02	− 0.001	− 0.07	0.01	–								
6. FFMQ Describe	29.50 (6.21)	− 0.38**	− 0.23*	− 0.33**	− 0.09	0.23*	–							
7. FFMQ Act Aware	28.23 (6.18)	− 0.55**	− 0.48**	− 0.43**	− 0.36**	0.07	0.56**	–						
8. FFMQ Nonjudge	29.37 (6.90)	− 0.53**	− 0.37**	− 0.54**	− 0.30*	− 0.001	0.46**	0.46**	–					
9. FFMQ Nonreact	22.70 (4.06)	− 0.38**	− 0.04	− 0.44**	− 0.17	0.31**	0.37**	0.24*	0.33**	–				
10. PBQ *wk*1	4.57 (4.31)	0.20*	0.21*	0.20*	0.21*	− 0.16	− 0.12	− 0.22*	− 0.27*	− 0.02	–			
11. PBQ *wk*3	5.03 (5.06)	0.20*	0.16	0.19	0.18	− 0.17	− 0.16	− 0.28*	− 0.37**	− 0.12	0.62**	–		
12. PBQ *wk*5	5.00 (4.97))	0.19	0.21	0.19	0.24*	− 0.10	− 0.13	− 0.29*	− 0.37**	− 0.10	0.60**	0.87**	–	
13. PBQ *wk*7	3.98 (3.82)	0.22*	0.24*	0.26*	0.21*	− 0.13	− 0.08	− 0.27*	− 0.29*	− 0.12	0.55**	0.80**	0.85**	–
14. Maternal age	30.75 (4.99)	− 0.19*	− 0.11	− 0.17	− 0.20	− 0.09	0.02	0.01	0.12	− 0.03	0.11	0.05	0.03	− 0.09
15. Education level	−	− 0.32**	0.08	− 0.27**	− 0.08	− 0.06	0.13	0.03	0.09	0.25**	0.16	0.13	0.17	0.10
16. Relationship status	−	− 0.03	− 0.10	− 0.03	− 0.08	− 0.19*	0.08	0.27**	0.11	− 0.11	− 0.05	0.03	0.01	0.11
17. Annual income	−	− 0.21*	0.003	− 0.15	− 0.02	0.11	0.03	− 0.06	− 0.02	0.10	0.06	0.10	0.13	0.09

*Note Ns* range from 94 to 117. IDAS = General Depression subscale of the Inventory of Depression and Anxiety Symptoms; GAD-7 = Generalized Anxiety Disorder-7; FFMQ = Five Facet Mindfulness Questionnaire (prenatal assessment); PBQ = Postpartum Bonding Questionnaire. *wk*1-*wk*7 = biweekly postnatal questionnaires completed four times during the first seven weeks

*p < .05, **p < .001

### Main analyses

#### Depression

In the model without interaction terms, prenatal depression was associated with bonding difficulties intercept (β_01_ = 0.10, *z* = 2.60, *p* = .009, 95% CI [0.02, 0.17]). No interaction was found between depression and the linear (β_11_ = 0.003, *z* = 0.09, *p* = .930, 95% CI [-0.06, 0.07]) or quadratic (β_21_=-0.001, *z*=-0.11, *p* = .911, 95% CI [-0.02, 0.02]) trends of time. The interactions between depression and the linear (β_11_=-0.05, *z*=-1.06, *p* = .289, 95% CI [-0.16, 0.05]) and quadratic (β_21_ = 0.01, *z* = 0.63, *p* = .528, 95% CI [-0.02, 0.04]) trends did not change after covarying for postnatal depression and the main effect of prenatal depression remained. Participants with higher prenatal depression showed elevated bonding difficulties at any time point, although depression was unrelated to the trajectory of bonding scores.

#### Anxiety

Greater prenatal anxiety predicted increased bonding difficulties (β_01_ = 0.27, *z* = 2.67, *p* = .008, 95% CI [0.07, 0.47]) but did not moderate the linear (β_11_=-0.007, *z*=-0.07, *p* = .942, 95% CI [-0.19, 0.17]) or quadratic (β_21_=-0.00004, *z*=-0.001, *p* = .999, 95% CI [-0.06, 0.06]) trends of time. Covarying for postnatal anxiety did not affect the significance of interactions with the linear (β_11_=-0.04, *z*=-0.35, *p* = .723, 95% CI [-0.27, 0.19]) or quadratic (β_21_ = 0.007, *z* = 0.19, *p* = .849, 95% CI [-0.07, 0.08]) trends and the main effect of prenatal anxiety remained. Participants with higher prenatal anxiety showed elevated bonding difficulties at any time point, although anxiety was unrelated to the trajectory of bonding scores.

#### Mindfulness

**Fig. 2:**
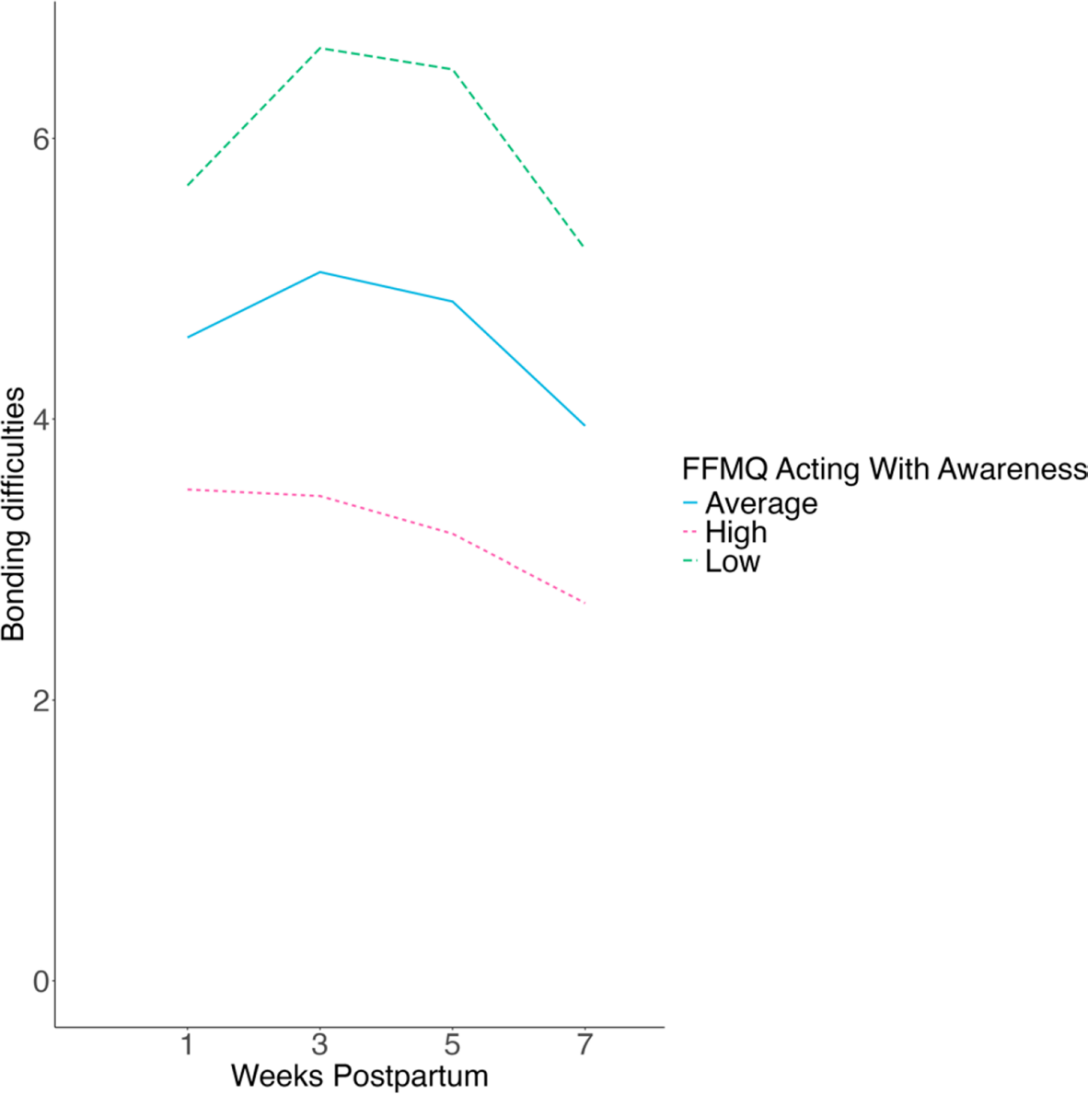
Simple slopes of the interaction between mothers’ tendency to act with awareness during pregnancy and time to predict maternal bonding difficulties across the first seven weeks postnatal. *Note* Average (blue) = at the mean on acting with awareness; High (pink) = one standard deviation above the mean on acting with awareness; Low (green) = one standard deviation below the mean on acting with awareness. At one standard deviation above the mean, the linear effect of time on bonding difficulties was not statistically significant (π_1j=_0.06, *z* = 0.12, *p* = .91, 95% CI [-0.97, 1.09]) and the quadratic effect was also not statistically significant (π_2j=_-0.11, *z*=-0.66, *p* = .51, 95% CI [-0.44, 0.22]). At one standard deviation below the mean, both the linear (π_1j=_1.55, *z* = 3.15, *p* = .002, 95% CI [0.59, 2.51]) and quadratic time trends (π_2j=_-0.56, *z*=-3.68, *p* < .001, 95% CI [-0.87, − 0.26]) were statistically significant

**Fig. 3:**
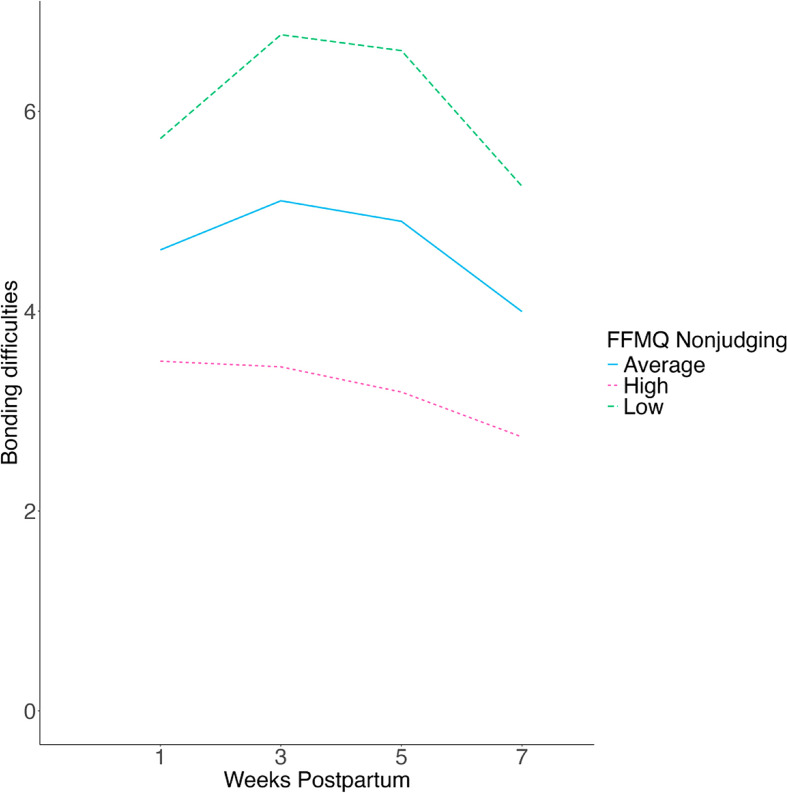
Simple slopes of the interaction between mothers’ tendency to be nonjudging of their own experience during pregnancy and time to predict maternal bonding difficulties across the first seven weeks postnatal. *Note* Average (blue) = at the mean on nonjudgment; High (pink) = one standard deviation above the mean on nonjudgment; Low (low) = one standard deviation below the mean on nonjudgment. At one standard deviation above the mean, neither the linear (π_1j=_-0.05, *z*=-0.10, *p* = .92, 95% CI [-1.05, 0.94]) nor quadratic time trend (π_2j=_-0.07, *z*=-0.43, *p* = .67, 95% CI [-0.38, 0.25]) were statistically significant. At one standard below the mean on nonjudging, both the linear (π_1j=_1.73, *z* = 3.50, *p* < .001, 95% CI [0.76, 2.70]) and quadratic trends (π_2j =_ -0.63, *z* = -4.00, *p* < .001, 95% CI [-0.94, − 0.32]) were statistically significant

Acting with awareness predicted lower bonding difficulties (β_01_=-0.23, *z*=-3.57, *p* < .001, 95% CI [-0.35, − 0.10]), weaker linear trend (β_11_=-0.12, *z*=-2.06, *p* = .040, 95% CI [-0.23, − 0.01]), and stronger quadratic trend (β_21_ = 0.04, *z* = 2.00, *p* = .046, 95% CI [0.001, 0.07]). These interactions were probed in simple slopes analyses presented in Fig. 2. Similarly, higher scores on the nonjudging facet predicted lower bonding difficulties (β_01_=-0.23, *z*=-4.20, *p* < .001, 95% CI [-0.34, − 0.12]), weaker linear trend (β_11_=-0.13, *z*=-2.52, *p* = .012, 95% CI − 0.23, − 0.03]), and stronger quadratic trend (β_21_ = 0.04, *z* = 2.49, *p* = .013, 95% CI [0.01, 0.07]). These interactions were probed in simple slopes analyses presented in Fig. 3. In contrast, facets of observing, describing, and nonreactivity were unrelated to bonding difficulties (see Supplementary Results).

## Discussion

The present study examined trajectories of maternal bonding difficulties across the first two months postnatal in 120 pregnant women oversampled for peripartum depression risk. We evaluated the role of prenatal internalizing symptoms and facets of mindfulness in predicting trajectories. Overall, results showed a trajectory of initial increases (worsening) in bonding difficulties that then stabilized before beginning to improve around five weeks postnatal and supported our a priori hypothesis that higher prenatal depression and anxiety would be associated with increased bonding difficulties across time. In turn, we found that greater prenatal mindfulness tendencies were associated with lower bonding difficulties across time and earlier improvements in feelings of connection.

The finding that bonding difficulties initially worsened before improving around five weeks postnatal has implications for our understanding of the development of feelings of bonding. Despite important conceptual and operational differences between maternal feelings of bonding and attachment representations (Bicking Kinsey and Hupcey 2013), results are consistent with attachment theory which suggests that the bond between caregiver and child strengthens over time (Bowlby 1969). Our close examination of the first two months postnatal suggests that the first three to five weeks postnatal may be challenging for some caregivers, displaying increased bonding difficulties that generally recovered with time. Caregivers may especially benefit from support and interventions in these early weeks, a period of adaptation when developing a bond with infants may be particularly important but difficult.

Second, symptoms of anxiety and depression during pregnancy were prospectively associated with increased bonding difficulties across time but did not interfere with the rate of change. These associations held when covarying for postnatal internalizing symptoms, highlighting the importance of considering prenatal symptoms when studying the onset of perinatal psychopathology and its implications for caregiving, despite most studies focusing on postnatal depression (Biaggi et al. 2016). Results follow contradicted literature: some previous studies documented that only prenatal anxiety, and not depressive symptoms, related to bonding difficulties at six weeks (Farré-Sender et al. 2018), and others, that prenatal depression, and not anxiety, was associated with bonding difficulties at three months (Dubber et al. 2015). Our results suggest that prenatal anxiety and depression are both risk markers for bonding difficulties across the early postnatal period. Importantly, our findings on the role of prenatal over postnatal symptoms suggest that the association is not only accounted for by current interference of symptoms with schemas and representations. They suggest that internalizing symptoms may also lead to compromised perceptions about self and others, including the child, lasting over the duration of the symptoms.

Third, results show that two specific mindfulness tendencies during pregnancy link to lower bonding difficulties: acting with awareness and being nonjudging of one’s thoughts and feelings. Similar results were obtained in previous research evaluating the associations between mindfulness facets using the FFMQ and pre- (acting with awareness, nonjudgment, and nonreactivity; Hicks et al. 2018) and postnatal (acting with awareness; Brassel et al. 2020) feelings of bonding towards the (unborn) baby. Given we were not powered to test for three-way interactions between internalizing symptoms, time, and mindful tendencies, future research using larger samples should evaluate whether dispositional mindfulness promotes feelings of bonding in the face of symptoms. Still, our results are informative to tailor interventions aiming to promote caregiver well-being and prevent bonding difficulties.

### Strengths and limitations

This study has several strengths, such as the longitudinal design and repeated assessments of bonding, the multilevel modeling approach, and the evaluation of risk and protective factors for maternal bonding. However, strengths should be interpreted considering some limitations. First, despite the longitudinal design suggesting temporality, the study design does not allow to draw causal conclusions related to the role anxiety, depression, and mindfulness play in shaping maternal feelings of bond. Second, our sample size, though larger than prior studies, was not large enough to explore complex interactions between risk and protective factors that may interfere with maternal feelings of connection. Future studies may consider exploring reciprocal associations between depression, anxiety, and bonding across multiple timepoints during the pre- and postnatal period. Third, we relied on a maternal-reported measure of bonding difficulties for which mean levels were relatively low (but similar to other studies; Foster et al. 2018; Kerstis et al. 2016) and that focuses on maternal perceptions rather than dyadic relationships. Subjective feelings of connection may be more closely linked to internalizing symptomatology and our associations might not be observed using observational, expert-coded dyadic bond (e.g., Nath et al. 2019). However, the PBQ is the most frequently used and validated questionnaire to assess maternal bonding difficulties.

## Conclusion

The present study investigated maternal bonding difficulties across the early postnatal period. Findings highlight a pattern of initial bonding difficulties that increases and peaks between 3 and 5 weeks but is followed by improvements from 5 to 7 weeks, with prenatal anxiety and depressive symptoms being associated with increased difficulties over time. We found that two specific facets of mindfulness during pregnancy, acting with awareness and being nonjudging of one’s own experience, were associated with greater feelings of connection with infants across the early postnatal. Overall, findings have empirical and clinical implications for our understanding of the development of maternal bonding and the early factors, present even before childbirth, that may be targeted by interventions aiming to support early postnatal adaptation and the caregiver−child relationship.

## Electronic supplementary material

Below is the link to the electronic supplementary material.

Supplementary Material 1

## Data Availability

De-identified data and code for this study are available by emailing the corresponding author.
