# Supplementary material for: Longitudinal associations between prenatal internalizing symptoms and mindfulness traits with postnatal bonding difficulties
Source: Arch Womens Ment Health. 2024 Sep 24;28(3):583–92. doi: 10.1007/s00737-024-01518-1 (PMC12092550; doi:10.1007/s00737-024-01518-1)
Supplement: Supplementary file 1 — Supplementary Material 1 [file 737_2024_1518_MOESM1_ESM.docx]

**Supplement for *Longitudinal associations between prenatal internalizing symptoms and mindfulness traits with postpartum bonding difficulties***

**Supplementary Methods**

***Participants and procedure***

In line with the main goals of the overarching study (i.e., longitudinal study on the predictors of postpartum depression), we oversampled participants with histories of lifetime depressive disorders to reach a sample at heightened risk for peripartum depression. We assessed depressive history with the Diagnostic Interview for Anxiety, Mood, and OCD and related Neuropsychiatric Disorders (DIAMOND; Tolin et al., 2018). At the prenatal assessment, half of the participants (51%, n = 61) met criteria for a past depressive disorder (i.e., major depressive disorder or persistent depressive disorder), and 14% met criteria for a current depressive (4%, n = 5) or anxiety (10%, n = 12) disorder.

Participants completed one prenatal assessment at mid gestation 20 weeks (mean=21.80, SD=3.16, range=17-29 weeks). The following timepoints were coordinated with the laboratory visits of the parent study, which included a final lab visit at 8 weeks postnatal. We thus planned four evenly spaced assessments between birth and the final study visit, i.e., at weeks 1, 3, 5, and 7 postnatal, to assess bonding difficulties. To do so, starting at 38 weeks’ gestation, participants received weekly text messages to confirm whether they had given birth. Once birth was confirmed, participants received a link via email to a first postnatal survey. The remaining three surveys were scheduled every two weeks such that participants completed four equally spaced postnatal surveys from approximately 1 week after birth (mean=0.72 weeks, SD=0.54) to 7 (mean=6.98 weeks, SD=1.05) weeks postpartum (*wk*1-*wk*7). Participants also completed a postnatal battery of assessments at 9 weeks postpartum (mean=9.23, SD=3.16, range=7-22 weeks).

**Supplementary Results**

***Test of shared method variance***

We conducted Harman’s single factor test including all items of our measures to evaluate the degree to which results may be a consequence of shared method variable. The unrotated solution produced a factor accounting for only 23% of the variance, i.e., below the threshold of 50% or more (Fuller et al., 2016; Kock et al., 2021), indicating that method bias is unlikely.

***Mindfulness facets of observing, describing and nonreactivity and postpartum bonding difficulties trajectories***

The mindfulness facet observing was not a statistically significant predictor of the intercept (β_01_=-.12, *z*=-1.61, *p*=.11, 95% CI [-.27, .03]), linear trend (β_11_=-.02, *z*=-.24, *p*=.81, 95% CI [-.15, .12]), or quadratic trend of bonding difficulties (β_21_=.01, *z*=.45, *p*=.65, 95% CI [-.03, .05]). Similarly, the describing facet of mindfulness was not a statistically significant predictor of the intercept (β_01_=-.10, *z*=-1.60, *p*=.11, 95% CI [-.23, .02]), linear (β_11_=-.05, *z*=-.87, *p*=.38, 95% CI [-.16, .06]), or quadratic trends (β_21_=.01, *z*=.84, *p*=.40, 95% CI [-.02, .05]). The final mindfulness facet, nonreactivity, was not a statistically significant predictor of the intercept (β_01_=-.10, *z*=-.98, *p*=.33, 95% CI [-.30, .10]), linear trend (β_11_=-.09, *z*=-1.03, *p*=.31, 95% CI [-.26, .08]), or quadratic trend of bonding difficulties (β_21_=.02, *z*=.74, *p*=.46, 95% CI [-.03, .07]).

**References Included in the Supplement**

Fuller CM, Simmering MJ, Atinc G, Atinc Y, Babin BJ (2016) Common methods variance detection in business research. J Bus Res 69(8):3192-3198. doi:10.1016/j.jbusres.2015.12.008

Kock F, Berbekova A, Assaf AG (2021) Understanding and managing the threat of common method bias: Detection, prevention and control. Tour Manag *86*:104330 [doi:10.1016/j.tourman.2021.104330](https://doi.org/10.1016/j.tourman.2021.104330)
